# The Estrogen-Related Receptor Alpha Upregulates Secretin Expressions in Response to Hypertonicity and Angiotensin II Stimulation

**DOI:** 10.1371/journal.pone.0039913

**Published:** 2012-06-25

**Authors:** Vien H. Y. Lee, Ian P. Y. Lam, Hueng-Sik Choi, Billy K. C. Chow, Leo T. O. Lee

**Affiliations:** 1 School of Biological Sciences, The University of Hong Kong, Pokfulam, Hong Kong SAR, China; 2 Hormone Research Center, School of Biological Sciences and Technology, Chonnam National University, Gwangju, Republic of Korea; Ecole Normale Supérieure de Lyon, France

## Abstract

Osmoregulation via maintenance of water and salt homeostasis is a vital process. In the brain, a functional secretin (SCT) and secretin receptor (SCTR) axis has recently been shown to mediate central actions of angiotensin II (ANGII), including initiation of water intake and stimulation of vasopressin (VP) expression and release. In this report, we provide evidence that estrogen-related receptor α (ERRα, NR3B1), a transcription factor mainly involved in metabolism, acts as an upstream activator of the *SCT* gene. *In vitro* studies using mouse hypothalamic cell line N-42 show that ERRα upregulates *SCT* promoter and gene expression. More importantly, knockdown of endogenous ERRα abolishes *SCT* promoter activation in response to hypertonic and ANGII stimulations. In mouse brain, ERRα coexpresses with SCT in various osmoregulatory brain regions, including the lamina terminalis and the paraventricular nucleus of the hypothalamus, and its expression is induced by hyperosmotic and ANGII treatments. Based on our data, we propose that both the upregulation of *ERRα* and/or the increased binding of ERRα to the mouse *SCT* promoter are two possible mechanisms for the elevated SCT expression upon hyperosmolality and central ANGII stimulation.

## Introduction

Secretin (SCT) is a classical gastrointestinal hormone [1]. Beyond its best known actions on the regulation of bicarbonate, electrolytes and volume secretion from the pancreatic ductular epithelial cells, there is a growing body of evidence showing SCT as a neuropeptide in the CNS [2]–[5] and as an anti-diuretic hormone, SCT stimulate the process of renal water reabsorption in kidney by a VP-independent mechanisms [6]. SCT is found as a neurosecretory factor released from the neurohypophysis into circulation upon water deprivation or direct electrical stimulation of the paraventricular nuclei (PVN) [6], [7] to control water reabsorption in the kidney [5]. Recently, the roles of SCT in the brain in water regulation were reported, and SCT was found to have similar functions as the osmoregulatory peptide, angiotensin II (ANGII) [8]. In these studies, with the aid of the *SCTR^−/−^* and *SCT^−/−^* mice, it was found that the presence of a functional SCT/SCTR axis is a prerequisite for some actions of ANGII, including the upregulation of *VP* expression in the hypothalamic PVN and the release of VP protein from posterior pituitary into the circulation. In addition, SCT itself was found as a dipsogenic hormone that controls water intake behavior as well as mediates the dipsogenic effects of ANGII [8].

In specific brain regions, both plasma hypertonicity and circulating ANGII are able to stimulate the lamina terminalis, consisting of the subfornical organ (SFO), organum vasculosum of the lamina terminalis (OVLT) and median preoptic nucleus (MnPO), which are critical sites for regulating water homeostasis in rodents [9]–[14]. Not just that we found SCT and SCTR expression in these water regulation sites, we found also that SCT injected into the lateral ventricle of the brain could augment water drinking behavior, as well as *VP* expression and VP protein release. As ANGII via binding to AT_1_ receptor could stimulate *SCT* expression in SFO and OVLT [7], it is therefore possible that SCT mediates central actions of ANGII partly by controlling the expression of SCT gene in these osmoregulatory brain regions.

The human and rat *SCT* promoter have previously been characterized [15]–[17], while more recently, small heterodimer partner (SHP), an orphan nuclear receptor, was identified as a repressor of the mouse *SCT* gene by blocking NeuroD-mediated gene activation in response to bile acids in the mouse duodenum [18]. Nuclear receptor superfamily is a large group of transcription factors that are involved in regulation of various physiological processes, including metabolism, cell growth and differentiation, development, and homeostasis [19]. A recent study on the genetically modified mouse model lacking estrogen-related receptor α (ERRα) revealed its functions in the renin-angiotensin system (RAS) to regulate renal sodium and potassium homeostasis [20], indicating potential roles of ERRα in osmoregulation. ERRα belongs to the NR3B1 subfamily of the orphan nuclear receptors and is apparently ligand-independent [21]. ERRα is expressed with the highest levels in tissues with high energy demands such as heart, kidney and adipose tissues [22], [23]. ERRα recognizes the estrogen response element (ERE) in target genes as estrogen receptor (ER) does, but was found to have a higher preference for the ERE-half site or ERR response element, which is a single half-site preceded by the three nucleotides TNA [24], [25].

In the present study, we investigated a possible mechanism for the cross-interaction of SCT and ANGII central pathways in osmoregulation via ERRα. We found that ERRα co-expresses with SCT in various osmocenters in the CNS, and that its own expression is regulated by central ANGII and peripheral hyperosmotic stimulation. Using a hypothalamic cell model, ERRα was then found to interact specificity with an ERE-half site in the proximal region of the mouse *SCT* promoter to control SCT gene expression. After showing that SCT plays an indispensable role in mediating central effects of ANGII (7), the present study, thus, provide evidence to indicate that ERRα is one of the mechanisms that connects SCT with central effects of ANGII and/or plasma osmolality.

## Materials and Methods

### Constructs and siRNA expressing vectors

The mouse SCT core promoter construct (mSCTP) (from −27 to −399 bp relative to the start codon) was produced by 1) PCR amplification using mouse genomic DNA, 2) sequencing, and 3) subcloning into the pGL2-Basic vector. The mouse ERRα, ERRβ and ERRγ expression vectors were constructed using the pcDNA3 vector (Invitrogen, Carlsbad, CA). Site-directed mutants of the mSCTP promoter were constructed by a three-step mutagenesis method [26] using the mutagenic primers listed in Table 1. For gene silencing, two siRNAs specific for the mouse ERRα (siERRα-S1 and siERRα-S2) were constructed using oligos (pSi-ERRα S1 and pSi-ERRα S2; Table 1) and the p*Silencer* 4.1-CMV vector (*Hind III*/*Bam HI* sites; Ambion, Austin, TX). The siControl vector provided by the manufacturer was used as a negative control. Western blot analysis [18] was used to check the effectiveness of siRNA in silencing endogenous mouse ERRα. Briefly, N-42 cells were transfected with 2 µg of the siERRα-S1, siERRα-S2, siControl or pSilencer vector by GeneJuice (see later for transfection procedures). After 24 h, cells were lysed with RIPA buffer (50 mM Tris pH 7.4, 0.25% Na-deoxycholate, 1% NP-40, 150 mM NaCl and 1 mM EDTA), and cell lysates were separated on a 10% SDS-PAGE and transferred to a nitrocellulose membrane. After incubation with an anti-ERRα antibody (1∶500 dilution; Millipore [27]) or GAPDH antibody (loading control, 1∶1000; Cell signaling technology) at 4°C overnight, and subsequently by the horseradish peroxidase conjugated secondary antibody (1∶1000 dilution; Santa Cruz Biotechnology) at room temperature for 1 h, signals were detected by the Western Lightning plus-ECL Enhanced Chemiluminescence Substrate (PerkinElmer, Shelton, CT).

**Table 1:** Nucleotide sequences of siRNA oligos, EMSA oligos, real-time PCR and mutation analysis (mutated nucleotides are underlined) primers.

Primer	Sequence (5′- 3″)
siERRα-S1-S	GATCCCTTTGCCTTTCCCGGGCCCTTCAAGAGAGGGCCCGGGAAAGGCAAAGGGA
siERRα-S1-AS	AGCTTCCCTTTGCCTTTCCCGGGCCCTCTCTTGAAGGGCCCGGGAAAGGCAAAGG
siERRα-S2-S	GATCCTGCTGGACCTCTGGCTCTATTCAAGAGATACAGCCAGAGGTCCAGCAGGA
siERRα-S2-AS	AGCTTCCTGCTGGACCTCTGGCTGTATCTCTTGAATACAGCCAGAGGTCCAGCAG
GS-ERRα-S	GCCCCTGACCTTCCCGG
GS-ERRα-AS	CCGGGAAGGTCAGGGC
SCTP-F	ACTACTCCTCTAATCTCCCC
SCTP-R	GACAAATGCTGCAACTTCAG
GAPDH-F	TGTGTCCGTCGTGGATCTGA
GAPDH-R	CCTGCTTCACCACCTTCTTGAT
SCT-F	GGTGGAGGGCCTCTATCTTC
SCT-R	CCAGGCGCTTATAATGGTGT
ERRα-F	TTCGGCGACTGCAAGCTC
ERRα-R	CACAGCCTCAGCATCTTCAATG
S14-F	CAGGACCAAGACCCCTGGA
S14-R	ATCTTCATCCCAGAGCGAGC
M1	GAGGGACAGGAGACAGCCGGACGACAG
M2	GGTGGGCGGCCCCAGCCTTCCTGCAAT
M3	TGGGCGGCCTGATTCTTCCTGCAATCA
M4	GGTGCAGCATTTAGTACACCCAGAGCC

### Transient transfection and luciferase assay

The mouse hypothalamic cell line, N-42, was purchased from CELLutions Biosystems Inc (Toronto, Canada). Cells were cultured in DMEM (31600-034; Invitrogen) supplemented with 4.5 mg/ml D-glucose, 10% FBS (Hyclone, Logan, UT), and the antibiotics, 100 U/ml penicillin G and 100 µg/ml streptomycin (Invitrogen) at 37°C with 5% CO_2_. For transfection, N-42 cells were seeded onto 6-well plates (35 mm/well; Costar, San Diego, CA) at a density 2.0×10^5^ cells/well. After 2 d, at about 80% cell confluence, transient transfection was carried out using the GeneJuice transfection reagent (Novagen, Darmstadt, Germany) according to the manufacturer's instruction. For over-expression studies, the mouse SCT promoter (mSCTP) [15]–[17] luciferase construct (2 µg) was co-transfected with different amounts of the ERRα, ERRβ, or ERRγ expression vectors (0.5, 1.0, 2.0 µg), and pCMV-β-gal as an internal control (0.5 µg) per well. For gene silencing studies, cells were cotransfected with mSCTP luciferase construct (2 µg), pCMV-β-gal (0.5 µg) and various amounts of silencing or control vector (1 and 2 µg). Appropriate amount of the pcDNA3.1 was added so that the total amount of DNA used per well was the same. Cell extracts were harvested 48 h after transfection for luciferase and β-galactosidase assays. The promoter activities were measured in at least three independent experiments each in triplicate assays normalized with β-galactosidase activity. In saline treatment studies, 48 h after transfection, different concentrations of saline (25, 50, and 100 mM) were added to the cells for 8 h at 37°C with 5% CO_2_ for luciferase assay or RNA isolation.

### The SCT expression in N42 cells after saline and ANGII treatment

Cell was seeded in 6-well plates at a density 2.0×10^5^ cells/well. After 2 d, at which the cell confluence was about 80%, saline treatment was carried out. Different concentrations of saline were added to the cells so that the final concentrations of the medium were 0, 25, 50, and 100 mM. The plate was then incubated at 37°C with 5% CO2 for various time periods (2 h, 4 h or, 8 h) before harvest. For ANGII treatments, the culture mediums were replaced by the medium with ANGII in various final concentration (ranged from 10^−7^ to 10^−11^ M). The cells were collected for 8 h before the RNA isolation and protein extraction. Total RNA were isolated by TRIzol reagent (Invitrogen). The isolated RNA was reverse transcribed with oligo-dT primer and Superscript III reverse-transcriptase (Invitrogen). One-tenth of the first strand cDNAs was then used as the template in qRT-PCR for measuring mouse *GAPDH*, *SCT*, *ERRα*, and *S14* mRNA levels by the SYBR Green PCR Master Mix kit (Applied Biosystems, Foster city, CA) with specific primers (Table 1). The fluorescence signals were measured in real time during the extension step by the 7300 Real Time PCR System (Applied Biosystems). The threshold cycle (Ct) was defined as the fractional cycle number at which the fluorescence signal reached 10-fold standard deviation of the baseline (from cycles 2 to 10). The ratio change in the target gene relative to the GAPDH control gene was determined by the 2^−ΔΔ Ct^ method [28]. For western blot analysis, cells were lysed with RIPA and western blot analysis of ERRαwere performed as described above

### MTS Assay

The saline treatments for N-42 cells was performed based on previous studies [29], [30], Cell proliferation assays were performed using the CellTiter 96 Aqueous One Solution Cell Proliferation Assay kit (Promega, Madison, MI) according to the manufacturer's instructions. N-42 cells were seeded at an optimized density of 2000 cells per well in a 96-well plate and maintained at 37°C with 5% CO_2_. After 48 h when cell confluence was about 70%, different concentrations of saline (0, 50, 100, 150, 200, 300, 400 mM) were added for 8 h. After salt treatment, CellTiter 96 Aqueous One Solution Reagent was added for 2 h and absorbance at 490 nm, which is directly proportional to the number of living cells in the culture, was measured. Wells with plain medium with no cells were included as the blank and each sample was repeated at least 5 times in this study.

### Electrophoretic Mobility Shift Assay (EMSA)

Nuclear extracts from mouse hypothalamus were prepared as described before [31]. Oligonucleotides containing the putative ERE motif (GS-ERRα-S and GS-ERRα-AS; Table 1) were annealed, end-labeled with [γ-^32^P] ATP by a Ready-To-Go T4-polynucleotide kinase labeling kit (Amersham Biosciences, Pittsburgh, PA) and purified by a Microspin G-25 column (Amersham Biosciences). Binding reactions were carried out at room temperature for 15 min in a 20 µl reaction containing 10 mM Tris (pH 7.5), 50 mM NaCl, 2.5 mM MgCl_2_, 0.5 mM dithiothretiol, 4% glycerol, 2 µg poly(deoxyinosine∶deoxycytosine), and 1 ρmol radio-labeled probe. Free probes and bound probes were separated by electrophoresis in a 5% polyacrylamide gel [16], [32]. In the supershift assay, 2 µg antibody against ERRα or Ap1 (Santa Cruz Biotechnology, Santa Cruz, CA, USA) were included in the reaction mix.

### Chromatin Immunoprecipitation (ChIP) Assay

ChIP assays were performed as described previously [33]. The mouse hypothalamus was isolated and nuclear extracts were prepared as described in [31]. The DNA-protein complexes in N-42 cells and mouse hypothalamus were cross-linked with 1.42% formaldehyde and harvested in IP buffer (150 mM NaCl, 50 mM Tris-HCL (pH 7.5), 5 mM EDTA, 0.5% NP-40, 1% Triton X-100, 0.5 mM phenylmethylsulfonyl fluoride and 10 µg/ml leupeptin). To shear the chromatin into DNA fragments, samples were sonicated (Sonifier 450; Branson Ultrasonics, Danbury, CT) by 1s-long pulses for 15 times at 50% maximum power output for 2 rounds. The DNA-ERRα complex was immunoprecipitated with 2 µg rabbit anti-ERRα IgG (Upstate, Millipore, Billerica, MA) in ice bath followed by the addition of protein A-agarose (Cruz Biotechnology, Santa Cruz, CA). DNA was isolated in Chelex-100 slurry (BioRad, Hercules, CA) and the protein was removed by proteinase K digestion (0.2 µg/µl). The purified DNA was used for quantitative real-time PCR (qRT-PCR) with specific primers (SCTP-F and SCTP-R; Table 1), targeting the mSCTP. The relative occupancy of the immunoprecipitation factor at the locus was estimated by 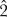 (Ct^mock^– Ct^specific^) and normalized with input DNA, where Ct^mock^ and Ct^specific^ represent the mean threshold cycles (Ct) of qRT-PCR (in triplicates) on purified DNA samples from mock and specific ERRα immunoprecipitation.

### Animal handling

All animal treatments were in accordance with the guidelines established by the Committee on the Use of Live Animals in Teaching and Research (CULATR, Approval ID 2072-10) of the University of Hong Kong with the Cap. 340 animal license issued by the Department of Health of the Hong Kong Government under the Animals Ordinance. All experiments were carried out with adult transgenic mice (20–25 g) of N5 generation, which were kept in a temperature-controlled room with a 12-h light/dark cycle. Mice were fed *ad libitum* with standard rodent chow (no. 5010, Test Diet, IN) and water, unless otherwise stated.

### Intracerebroventricular (ICV) incannulation and drug administration

Mice were anesthetized and surgery was conducted in aseptic conditions. Procedures for ICV were performed as described [34], [35]. For injections into the lateral ventricle, the coordinates of cannula implantation were determined according to the mouse brain atlas [36]. The cannula (11-mm-long, 21-gauge stainless steel tubing) was stereotaxically placed such that the tip was 0.5 mm caudal to bregma, 1.0 mm lateral to midline, and 2.0 mm below the dura. The placement was confirmed by injection of a dye. Three days were allowed for recovery before ICV injection. Central injections were done using PE-10 tubing attached to an injector and a 10-µl Hamilton syringe. Artificial cerebrospinal fluid (ACSF; according to Alzet protocol, 2 or 5 µl), ANGII (100 ng/2 µl; 002-12; Phoenix Pharmaceuticals, Belmont, CA), or saline (0.25 or 0.50 M/5 µl) were injected into the lateral ventricle.

### Water deprivation and saline drinking followed by Laser-capture microdissection (LCM) and qRT-PCR

To induce hypertonic serum in mice, normal drinking water was either removed for 1 d or replaced with 2% saline for 5 d. Treated and control mice were then sacrificed and their brains were collected. For ICV studies, 1 h after the ICV injection, similarly, brains of treated and control mice were isolated. For whole brain studies, total RNA was extracted by the TriPure isolation reagent (Roche Molecular Biochemicals, Basel, Switzerland). For study of the specific brain areas, the brains were embedded in OCT compound (Sarura Finetek, Torrance, CA) and stored at −20°C. The frozen brain was then sectioned (8-µm thick) using a cryostat microtome (Jung CM3000, Lieca Microsystems, Leitz, Germany) and mounted on plain slices. Brain sections at the level of the lamina terminalis and PVN were stained with hematoxylin and eosin and imaged on the PixCell IIe Laser Capture Microdissection System (Arcturus, Sunnyvale, CA). Target cells were located according to the mouse brain atlas and were captured on CapSure HS LCM Caps (Arcturus). RNA of captured cells was prepared using the PicoPure RNA Isolation Kit (Arcturus). The isolated RNA was reverse transcribed and real-time PCR were performed as described above.

### Immunohistochemical (IHC) staining

IHC staining was performed as described [6], [35]. Brains isolated from mice were fixed in 3.7% formalin, embedded in paraffin, and sectioned (7 µm). Paraffin sections were dewaxed and rehydrated in graded ethanol. Endogenous peroxidase activity was blocked by 3% hydrogen peroxide in methanol. Microwave antigen retrieval was performed with citric acid buffer at pH 6.0 for 10 min, followed by blocking of non-immunological binding with 5% normal goat serum for 2 h. Sections were then incubated with rabbit anti-ERRα IgG (1∶500 dilution; Millipore) overnight at 4°C. Primary antibody was replaced by 1×PBS in the negative controls. Immunoreactive signals were obtained by the Vectastain ABC Elite kit (Vector Laboratories, Burlingame, CA) in light brown color using 1× DAB substrate (Roche Diagnostics, Shanghai, China) and counterstained with hematoxylin (Zymed Laboratories, San Francisco, CA).

### Statistical analysis

All data are shown as means ± standard error SEM. The deviations between groups were analyzed using the computer software PRISM (version 3.0, GraphPad Software, La Jolla, CA). Unpaired t-test was performed when only two groups were under consideration, whereas data from more than two groups were analyzed by one-way ANOVA, followed by Dunnett's test.

## Results

### ERRα upregulates *SCT* gene in the hypothalamic N-42 cell line

**Figure 1:**
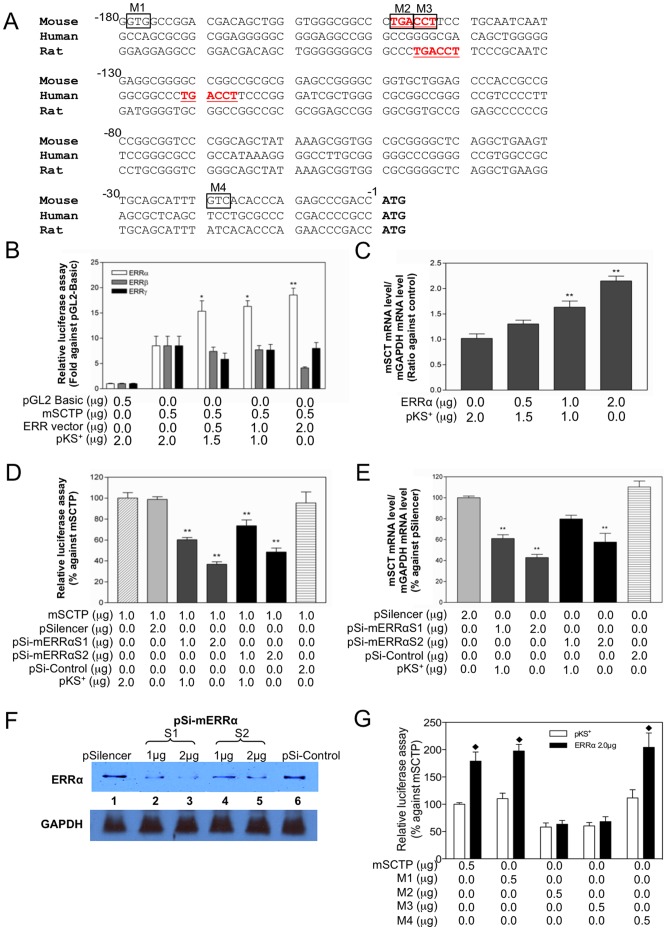
ERRα upregulates SCT expression in N-42 cells. (A) The 5′ upstream sequence of the *SCT* gene contains a ERE-half site. The nucleotide sequences (180 bp) upstream of the start codon of the mouse, human and rat *SCT* gene are shown. The first nucleotide of the start codon (ATG) is assigned as +1. The consensus sequences of the putative ERE-half site are highlighted in red and underlined. The mutation sites are indicated by the boxes (B) Effects of over-expressing ERRs in N-42 on the mouse *SCT* promoter. mSCTP (0.5 µg) and various amounts of the 3 isoforms of ERR/pcDNA3 (0, 0.5, 1.0 and 2.0 µg) were cotransfected into N-42 cells. Total DNA was adjusted to 2.5 µg by pKS^+^. *p<0.05; **p<0.001, compared with mSCTP (0.5 µg). (C) Effects of over-expressing ERRα on the mouse SCT mRNA levels in N-42 cells. The mRNA levels of mouse *SCT* measured by real-time PCR were normalized with mouse *GAPDH* levels. *p<0.05; **p<0.001, compared with control (ERRα – 0 µg). (D–E) Effects of endogenous silencing of mouse ERRα on (D) mouse SCT promoter and (E) mRNA levels in N-42 cells. The mSCTP (1.0 µg) was co-transfected with various amounts of siERRα-1 and siERRα-2 (1.0 and 2.0 µg), pSilencer or siControl into N-42 cells. The mRNA levels of mouse *SCT* measured by real-time PCR were normalized with mouse *GAPDH* levels. Data represent the mean ± SEM of three experiments performed in duplicates. *p<0.05; **p<0.001, compared with mSCTP – 1.0 µg. (B) *p<0.05; **p<0.001, compared with control (pSilencer – 2.0 µg). (F) Western blot analysis of ERRα protein in N-42 cells transfected with (1) pSilencer (2.0 µg), (2) siERRα-S1, (3) siERRα-S2 and (4) siControl (2.0 µg). The GAPDH western blot was used as the loading control. (G) Mutation analysis of ERE-half site. Four mutants (M1–M4, 0.5 µg) were cotransfected with pKS^+^ or ERRα expression vector (2.0 µg). ⧫p<0.05; ERRα cotransfected promoter compared with the same construct that transfected with pKS^+^ (0.5 µg).

In view of a recent study revealing a potential function of ERRα in the renin-angiotensin system (RAS) for osmoregulation [9] and the presence of an ERE-half site in the proximal region of the mouse (from −149 to −144 bp, relative to the start codon), rat and human *SCT* promoters (Fig. 1A), we initially investigated the *in vitro* function of ERRs to control the mouse *SCT* gene in a mouse hypothalamic N-42 cell-line. In this study, each of the three isoforms of ERR (α, β and γ) was overexpressed with the mouse *SCT* promoter-luciferase construct. As shown in Fig. 1B, the *SCT* promoter was significantly (1.92-fold and 2.18-fold for 1.0 and 2.0 µg of ERRα, respectively) activated only by ERRα, which is a constitutive activator [37], [38], whereas overexpression of neither ERRβ nor ERRγ showed an activation effect on mouse *SCT* promoter. Consistent with this observation, endogenous *SCT* mRNA levels were significantly elevated (1.63-fold and 2.15-fold for 1.0 and 2.0 µg of ERRα, respectively) when ERRα was overexpressed in N-42 cells (Fig. 1C). In order to confirm further a functional role of ERRα, two siRNA vectors specific for targeting the mouse *ERRα* were designed. Silencing of the endogenous ERRα in N-42 cells, by using either of the silencing vectors (S1 or S2), resulted in considerable drops in the mouse *SCT* promoter activities (S1: 39.7% and 63.2% decrease; S2: 26.4% and 51.6% decrease) (Fig. 1D), and also in the endogenous *SCT* transcript levels (S1: 38.9% and 57.2% decrease; S2: 42.4% decrease for 2 µg) (Fig. 1E). The siRNA were functional in lowering endogenous ERRα expression as indicated by Western blotting (Fig. 1F), in which ERRα protein levels were substantially reduced. These findings suggested that ERRα is responsible for the upregulation of mouse *SCT* expression in the hypothalamic cells. The direct relationship between the ERE-half site and the ERRα mediated activation of mSCTP were confirmed by mutation analysis (Fig. 1G). The promoter activities were increased with ERRα cotransfection in the mutants that not related to ERE-half site (M1 1.8 fold and M4 1.9 fold when compare to pKS+ control), whereas the mutation of ERE-half site can severely decrease ERRα-mediated activation effects (only 1.08 fold for M2 and 1.13 fold for M3). The data clearly suggested that the ERE-half site is the critical motif for the ERRα responsiveness in mSCTP.

### ERRα is a mediator of the hypertonicity- and ANGII-induced upregulation of *SCT*

**Figure 2:**
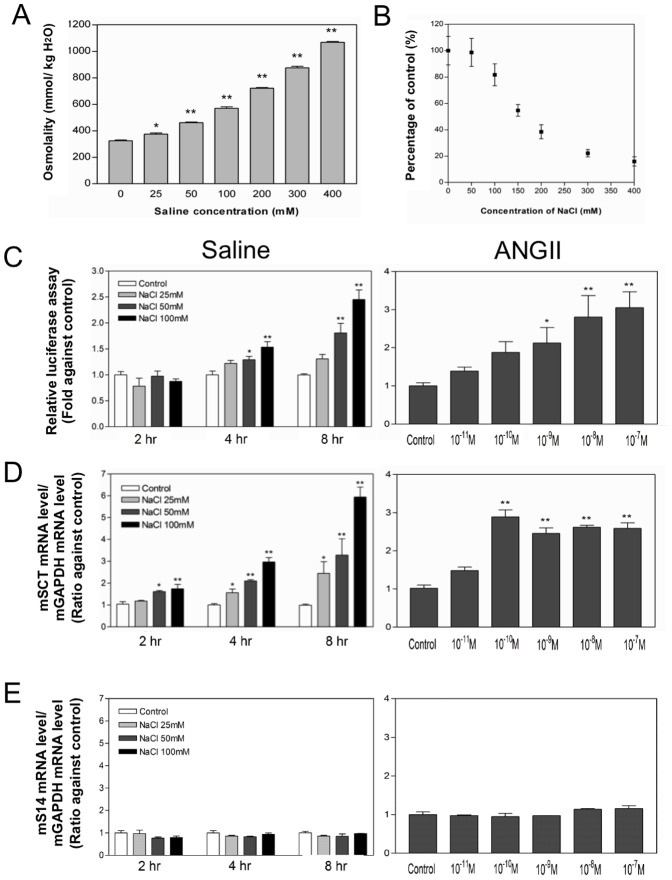
Effects of saline and ANGII treatments on *SCT* promoter activity and gene expression in N-42 cells. (A) Osmolality of culture medium after treatment of different concentrations of saline (0, 50, 100, 200, 300, 400 mM) for 8 h in N-42 cells. (B) Percentage of cells surviving after saline treatments with medium of various concentrations (0, 50, 100, 150, 200, 300, 400 mM) for 8 h in N-42 cells. (C) Cells were transfected with mSCTP (2.0 µg) for 2 d and treated with various saline concentrations (25, 50, 100 mM) for different times (2, 4, 8 h) or ANGII (10^−10^ to 10^−7^ M) for 8 h. Data represent the mean ± SEM of three experiments performed in triplicates. (D and E) Cells were treated with various saline concentrations (25, 50, 100 mM) for different times (2, 4, 8 h) and RNA was extracted afterwards. The mRNA levels of mouse *SCT* (D) and *S14* (E) were normalized with mouse *GAPDH* levels. Data represent the mean ± SEM of three experiments performed in duplicates.*p<0.05; **p<0.001, compared with the respective control.

After showing ERRα as an activator of the *SCT* gene, our next task was to study if ERRα was involved also in the osmoregulatory pathway. We have previously revealed that *SCT* expression was upregulated in the mouse hypothalamus in response to water deprivation and saline drinking [8]. To examine the effect of hypertonic shock on the mouse *SCT* gene, N-42 cells were treated with different concentrations of hypertonic salt treatment (25, 50 and 100 mM) for 2, 4 and 8 h (Fig. 2C) as in a previous study [29]. Before the treatment, the toxicity of hypertonic saline to the N42 cells was tested by MTS assay. The corresponding osmolality in different concentration of NaCl was shown in Fig. 2A. The MTS assay (Fig. 2B) suggested that the lethal concentration 50 of NaCl is around 150 mM. Therefore, the maximum concentration of NaCl that used in the N42 cells treatment is 100 mM. Hypertonic treatment of cells led to time- and dose-dependent increases in *SCT* promoter activities (Fig. 2C), in which the 8-h treatment with 100 mM saline resulted in the strongest induction (2.45-fold). Likewise, hypertonic saline treatment could stimulate endogenous *SCT* expression in a similar pattern (Fig. 2D). In this study, the mouse house-keeping gene *S14* was used as a control which expression was not affected by hypertonic saline under all the conditions tested (Fig. 2E). Consistently, ANGII peptide dose-dependently (10^−11^ to 10^−7^ M, 8 h treatment) activated mouse *SCT* promoter (Fig. 2C) as well as endogenous *SCT* mRNA levels (Fig. 2D). ANGII at the concentration 10^−10^ M was found to produce the greatest stimulation (2.89 folds). Again, ANGII treatment was unable to change the expression of the mouse *S14* housekeeping gene, as a control.

**Figure 3:**
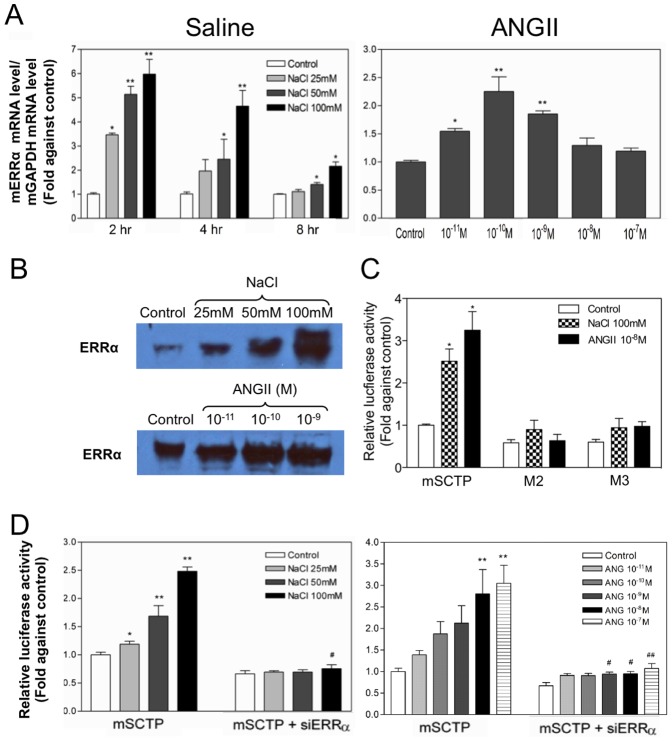
Effects of ERRα silencing on the *SCT* promoter in response to hyperosmotic shock. (A) Cells were treated with various saline concentrations (25, 50, 100 mM) for different times (2, 4, 8 h) or ANGII (10^−11^ to 10^−7^ M) for 8 h, and RNA was extracted afterwards. The mRNA levels of mouse *ERRα* were normalized with mouse *GAPDH* levels. Data represent the mean ± SEM of three experiments performed in duplicates.*p<0.05; **p<0.001, compared with the respective control. (B) Western blot analysis of ERRα protein in N-42 cells after saline (upper) and ANGII (10^−11^ to 10^−9^ M). (C) Effect of saline and ANGII treatment on ERE-half site mutants. Cells were transfected with mSCTP, M2 or M3 (2.0 µg) for 2 d and treated with saline (100 mM) for different times or ANGII (10^−8^ M) for 8 h. Data represent the mean ± SEM of three experiments performed in triplicates. ^*^p<0.05, compared with the control promoter without treatment. (D) N-42 cells were transfected with mSCTP (2.0 µg) and either pSilencer (Control) (2.0 µg) or pSi-mERRα-S1 (2.0 µg). After 2 d, cells were subject to treatement with saline-added medium of different concentrations (25, 50, 100 mM) or ANGII (10^−10^ to 10^−7^ M) for 8 h. Data represent the mean ± SEM of three experiments performed in triplicates.*p<0.05; **p<0.001, compared with the control transfected with pSilencer without treatment. ^#^p<0.05, compared with the control transfected with pSi-mERRα-S1 without treatment.

Similar to their effects on *SCT*, both hypertonic shock and ANGII could also stimulate mouse *ERRα* mRNA expression (Fig. 3A) as well as the ERR*α* protein level (Fig. 3B) in this hypothalamic cell model. When siERRα construct (pSi-m*ERRα*-S1) was employed to silence endogenous ERRα, consistent with findings in Fig. 2D, the effects of both hypertonic saline and ANGII treatment were almost completely abolished (Figure 3D). This upregulation of mouse SCT expression is dependent on the ERE-half site. As shown in Fig. 3C, the mutation of this motif (M2 and M3) cause a large and significant reduction in the response to the saline and ANGII treatment. Taken together, our data show the potential function of ERRα in mediating stimulatory effects of hypertonic saline and ANGII on *SCT* expression.

### *In vivo* functions of ERRα in osmoregulation in the mouse brains

**Figure 4:**
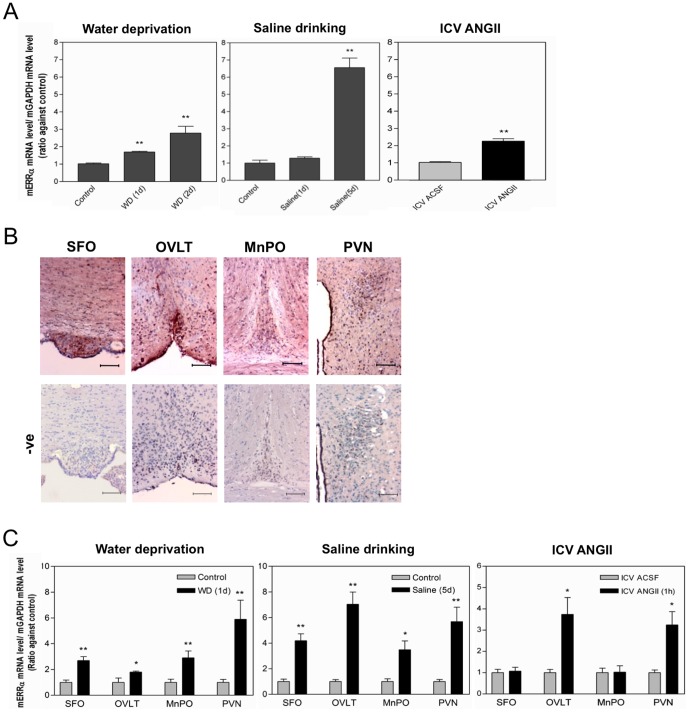
Effects of water deprivation, saline dinking and central ANGII administration on mouse *ERRα* expressions in mouse brain. Mice were water deprived, provided with hypertonic saline (2%) for 1 or 5 d, or centrally injected with ANGII peptide. The mRNA levels of mouse *ERR*α in (A) the whole mouse brain or (C) the isolated osmosentitive brain regions SFO, OVLT, MnPO and PVN, were measured by real-time PCR were normalized with mouse *GAPDH* levels. Data are expressed as the mean ± SEM (n = 10/group). *p<0.05; **p<0.001, compared with control. (B) Immunohistochemical staining showing ERRα immunoreactivities in the SFO, MnPO, OVLT and PVN of the WT mouse brain. Negative control was done by using 1× PBS instead of the primary anti-ERRα antibody. Bars, 6 µm.

To study the *in vivo* functions of *ERRα* to upregulate *SCT* in response to hypertonic treatment and ANGII, we initially investigated expression levels of *ERRα* in mouse brain in response to hyperosmotic stimuli and central ANGII administration. We found that *ERRα* transcript levels in the mouse cerebrum were significantly elevated after water deprivation, saline drinking or ICV-ANGII in mice (Fig. 4A). We have previously shown the localization of SCT transcript and protein in various osmoregulatory sites, including the SFO, OVLT and MnPO, and that *SCT* transcript levels were significantly enhanced upon water deprivation and saline drinking [8]. By IHC stainings, ERRα proteins were also found in sites collectively known as lamina terminalis and PVN of the mouse brain (Fig. 4B). By LCM-coupled with qRT-PCR, expression changes of *ERRα* in these brain regions were studied (Fig. 4C). *ERRα* transcript levels in the PVN, SFO, MnPO and OVLT were significantly augmented after water deprivation and saline drinking (Fig. 4C). One hour after ICV-ANGII injection, *ERRα* levels in the PVN and OVLT were elevated while its levels in the SFO and MnPO were unchanged (Fig. 4C) The expression changes of ERRα could therefore activate SCT expression in these areas as shown in our in vitro studies in N42 cells.

### *In vitro* and *in vivo* binding of ERRα to the ERE-half site of the mouse *SCT* promoter

**Figure 5:**
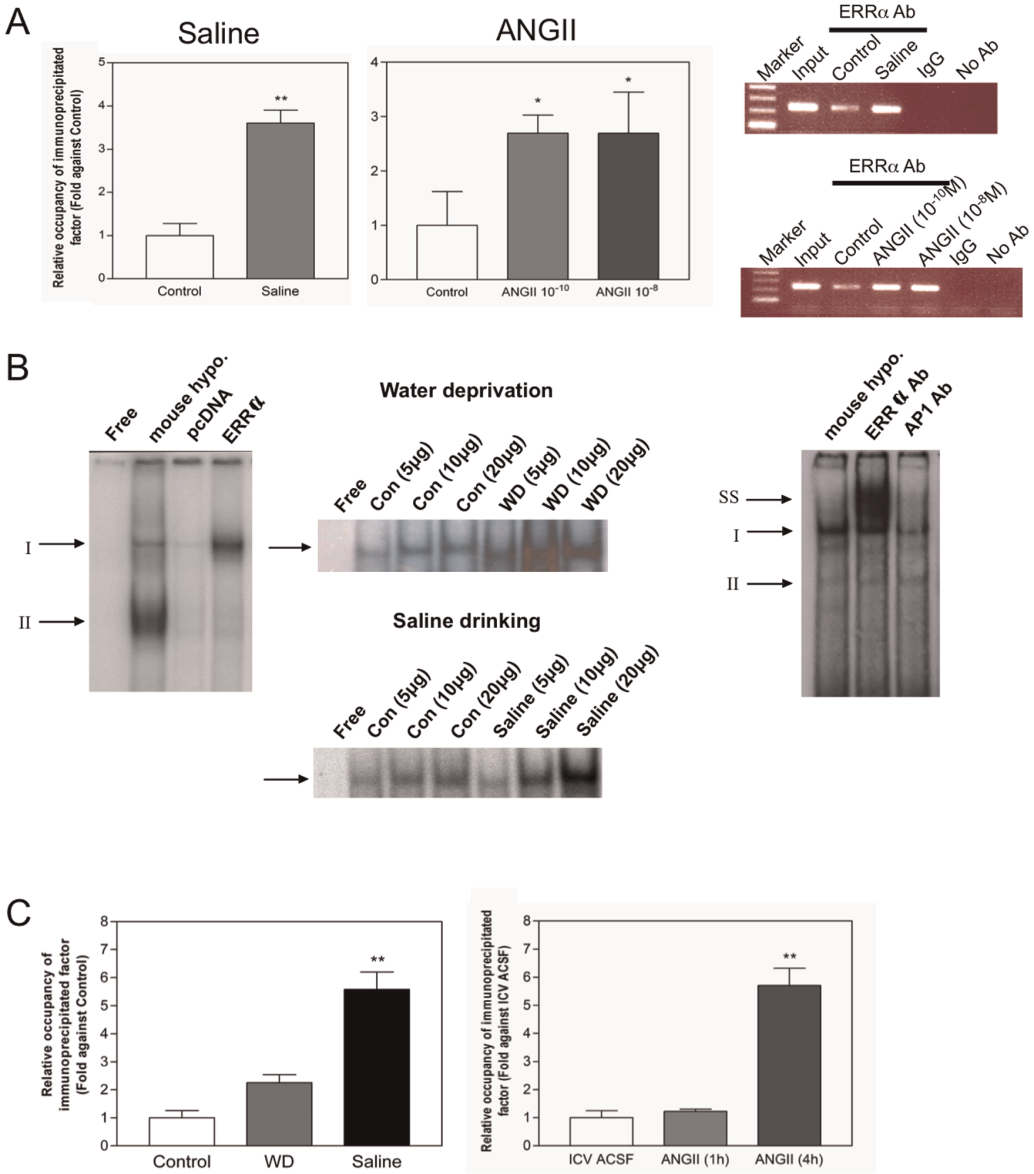
Effect of saline and ANGII on the binding of ERRα to the mouse SCT promoter. (A) ChIP assay showing the relative occupancy of the immunoprecipitated ERRα at the mouse *SCT* promoter in N42 cells after saline and ANGII treatment for 8 h in N-42 cells. Data was calculated using the equation: 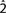 (Ct^mock^–Ct^specific^) and normalized with Input, which was defined as 1.00. A mock immunoprecipitation using rabbit anti-IgG and without using antibody were carried out as negative control. Data represent the mean ± SEM of three experiments.*p<0.05; **p<0.001, compared with the control. (B) EMSA using the ERE-half site as the oligo. Left: Nuclear extract of mouse hypothalamus (10 µg), pcDNA (control), and *in vitro* translated ERRα protein were pre-incubated with the oligo for 15 minutes at room temperature. Arrows indicate specific protein-DNA complexes that contain ERRα (Complex I) and unknown protein (complex II). Middle: Hypothalamic nuclear extract of control or water deprived or saline-drank mice were used (n = 4/group) in EMSA. Right: Supershift assay of ERRα proteins. Antibodies (2 µg) specific for ERRα and AP1 (Santa Cruz) proteins were pre-incubated with the nuclear extract (10 µg) for 20 min at room temperature. Arrows indicate specific protein–DNA complexes that contain ERRα (Complex I), non-specific complex (Complexes II) and ERRα supershift (SS). (C) ChIP assay showing the in vivo binding of ERRα on mouse *SCT* promoter in mouse hypothalamus after water deprivation and saline treatment (left) and ICV-ANGII injection in mice (1 and 4 h) (right).

Despite the presence of an ERE-half site in the mouse *SCT* promoter (Fig. 1A), there was no previous report showing the functional interaction of ERRα with this motif. In the present study, using EMSA and ChIP assay, we seek to investigate the binding of ERRα to the *SCT* promoter ERE-half site in N-42 cells and mouse hypothalamus (Fig. 5). In the ChIP assay (Fig. 5A), we observed no PCR signal from the negative controls including the no antibody, and anti-rabbit IgG control. The controls show neither nonspecific precipitations nor PCR contamination in this assay. Positive PCR signals are found in the samples treated with ERRα antibody. This suggested the *in vivo* binding of ERRα with the mouse *SCT* promoter. The ChIP assay coupled to qRT-PCR, more importantly, showed that both saline and ANGII treatments were able to increase the ERRα binding. EMSA using mouse hypothalamic nuclear extract showed the presence of two DNA-protein complexes (I and II) (Fig. 5B left). To confirm the identity of these complexes, we performed EMSA by incubating the same probe with *in vitro* translated mouse ERRα proteins, which was found to form a complex with a size similar to complex I. In the ERRα supershift assay, the intensity of complex I was decreased and a supershift band was observed (Fig. 5B right). No supershift band can be observed in the control (AP1 antibody). This supershift assays suggests that complex I contains ERRα protein. To show changes in binding of ERRα, we have used nuclear extracts prepared from the hypothalamus of mice under water deprivation and saline drinking, and we found that the intensities of complex I were increased after these treatments (Fig. 5B middle).

Consistent with results in these *in vitro* studies (Fig. 5A), ChIP assay showed the *in vivo* occupancy of the *SCT* ERE-half site with ERRα was greatly induced by water deprivation, saline drinking, and 4 h after the ICV-ANGII administration (5.83-fold) in the mouse hypothalamus (Fig. 5C). In summary, the above findings indicate the increased expression and binding of ERRα with the *SCT* ERE-half site in the mouse hypothalamus is a mechanism for the elevated *SCT* expressions in response to hyperosmolality and central ANGII stimulation.

## Discussion

By reviewing the 5′ sequence (1800 bp upstream of the transcriptional start site) of the mouse *SCT* gene, an ERE-half site, a putative binding site for the orphan nuclear receptor ERR, is present at −149 bp (Fig. 1A). In the past, there were several studies revealing essential regulatory sequence motifs within the human and rat *SCT* promoter [15], [16], [39], and recently, the mouse *SCT* gene has been shown to be regulated by Sp1/Sp3 interacting with two GC-boxes (at −125 and −158) and NeuroD binding with an E-box motif (at −167), [17], [18]. In the present study, we provided evidences that ERRα interacting with the ERE-half site in the mouse *SCT* promoter is implicated for mediating central effects of ANGII.

There have been studies suggesting estrogen plays functional roles in fluid regulation in rodent and human [40]–[42]. ERRs are closely related to ERs, with which they share almost identical DBD (68% amino acid homology) [43]–[45]. In view of its close relationship with the ER, and that ERRα is often shown to act synergistically with ERα [25], ERRα is hypothesized as a possible candidate that participates in osmoregulation. In the present study, ERRα expression was revealed to be responsive to ANGII stimulation in the hypothalamic cells, and in the lamina terminalis and PVN of mouse brain. This is similar to previous study on ERα that water deprivation led to increased ERα expression in the SFO [46]. ERα is localized extensively in the osmosensitive neurons in the lamina terminalis and the SON, whereas ERβ is prominently expressed in the VP magnocellular neurons of rat hypothalamus [47], [48]. Likewise, using immunohistochemical staining and real-time PCR, the present study showed that ERRα proteins and transcripts were localized in the lamina terminalis and PVN, which are important brain regions for the control of VP secretion and drinking behavior.

In agreement with its expression patterns in high energy demand tissues, ERRα is generally recognized as a transcription factor that regulates expression of genes involved in bioenergetics [49], [50], as a biomarker for several human cancers [51]–[53], bone homeostasis [54], as well as in cell proliferation and migration [55], [56]. The ERRα knockout mice are viable, fertile and display no gross anatomical alterations, but have reduced body weight with less peripheral fat mass [57]. More recently, a physiological study of ERRα deficient (*ERRα^−/−^*) mice have identified the potential of ERRα in regulating blood pressure and water homeostasis, by controlling renal sodium and potassium homeostasis and the RAS [20]. In summary, phenotypic analysis revealed that the *ERRα^−/−^* mice have reduced water intake, urine volume, and increased Na^+^ in blood. These indicate that ERRα is dipsogenic and diuretic. Also, ERRα knockout mice are deficient in renal Na^+^/K^+^ handling with a mechanism that favor Na^+^ retention, leading to the increased blood Na^+^ and thus increased water reabsorption and decreased urine volume. Using genome-wide ChIP-on-ChIP analysis and expression profile comparison of *ERRα^−/−^* and WT mice, the study also identified a number of target genes regulated by ERRα in kidney. Some of these are genes involved in water regulation, including the RAS genes (*Ren1*, *Agt*, *Ace2*), VP receptor (*Avpr1a*, *Avpr2*), and tonicity-responsive element (TonE) binding protein (*TonEBP*). Our findings show that ANGII upregulates *ERRα* in the lamina terminalis and PVN in the brain; while Tremblay *et al.* suggested that ERRα could downregulate RAS genes in kidney, which might work as a negative feedback control [20]. Compatible with our study that shows ANGII can stimulate *ERRα*, and thus *SCT* and *VP* expression; VP receptor genes in kidney were found to be downregulated in the *ERRα^−/−^* mice [20], indicating that ERRα is responsible for intensifying the renal actions of VP. In summary, this report suggests new functions of ERRα in osmoregulation by regulating SCT's action in the brain. In view of an indispensable role of SCT and SCTR axis in mediating functions of ANGII in the central nervous system, this study not only confirmed, but also filled in the missing gap between ANGII and SCT in this newly discovered osmoregulatory pathway.
